# 4207 Development and Evaluation of a Pilot Mentor Training Program for Clinical Translational Research Professional Workforce

**DOI:** 10.1017/cts.2020.208

**Published:** 2020-07-29

**Authors:** Yulia A. Strekalova, H. Robert Kolb, Holly Morris, Rebecca Austin-Datta

**Affiliations:** 1 University of Florida; 2 University of Florida CTSI

## Abstract

OBJECTIVES/GOALS: The goal of this project was to develop and evaluate a pilot mentor training program for clinical research professionals. This project presents an evidence- and theory-based mentoring program that has been developed, implemented, and evaluated for this group of translational research professions. METHODS/STUDY POPULATION: The curriculum for the program was designed for aspiring mentors and aligned with the topics of existing Entering Mentoring curriculum for translational workforce (Pfund, Branchaw & Handelsman, 2015). Eleven experienced CRPs participated in the pilot training program. The training was delivered in two-hour meetings over eight weeks. Qualitative e-mail interviews and a validated mentoring competency assessment (Fleming et al., 2013) and mentor role assessment (Dilmore, 2010) tool were used for process and outcome evaluation. Cases studies specific to the CRPs work environment were developed and used to facilitate discussions throughout the training. RESULTS/ANTICIPATED RESULTS: Pre- and post-training scores for mentoring competency assessment were compared across six sub-indexes. Paired t-tests showed a significant difference for the maintaining effective communication competency, p = 0.0202. Comparisons of individual items also showed positive changes in the promoting professional development competency, p = 0.0161). Qualitative assessment revealed that most mentor trainees recognized a distinction between a mentor and a supervisor or on-the-job-trainer. Furthermore, most have been informal mentors without a formal role assignment, the need for ongoing mentoring, and potential of mentoring networks. DISCUSSION/SIGNIFICANCE OF IMPACT: CRPs is a diverse group of research support professionals who may hold the roles of research study coordinators, research nurses, regulatory and compliance specialists. Tailored mentoring can provide essential infrastructure for ongoing professional development and support talent retention.

